# Tracheal stenosis following tracheotomy in a COVID‐19 patient

**DOI:** 10.1002/rcr2.1127

**Published:** 2023-03-26

**Authors:** Mohamed Tayeb Salaouatchi, Linda Spinato, Said Sanoussi, Maria do Carmo Filomena Mesquita

**Affiliations:** ^1^ Centre Hospitalier Universitaire Brugmann, Place A.Van Gehuchten 4 1020 Bruxelles Belgium

**Keywords:** ARDS, COVID‐19, mechanical ventilation, post tracheotomy stenosis, stridor

## Abstract

Hemodialyzed patients with COVID‐19 are at risk for severe complications from acute respiratory distress syndrome, requiring admission to the intensive‐care unit for invasive mechanical ventilation. Post tracheotomy stenosis can be a life‐threatening condition that commonly occurs after iatrogenic injury secondary to tracheotomy or tracheal intubation. We report a case of a 44‐year‐old female patient on maintenance haemodialysis who presented a COVID‐19‐related ARDS that required mechanical ventilation for 4 weeks, followed by a persistent stridor and finally succumbed, 1 month after being discharged from intensive care unit, from a severe respiratory distress due to a tracheal stenosis. Our aim is to highlight the importance of the early recognition and management of post tracheotomy stenosis in patients with persistent respiratory difficulty as stridor after prolonged intubation requiring tracheotomy, in order to improve the prognosis of these patients.

## Graphical abstract

We highlight the importance of the early recognition and management of post tracheotomy tracheal stenosis in patients with persistent respiratory difficulty as stridor in order to improve the management and prognosis of these patients.

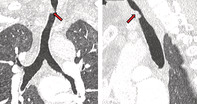

## INTRODUCTION

Patients with end‐stage‐renal‐disease (ESRD) undergoing haemodialysis (HD) represent a vulnerable population for severe acute respiratory syndrome coronavirus 2 (SARS‐CoV‐2) infection. Intensive care unit (ICU) admission and mortality rates were reported to be significantly higher in groups with kidney disease, including patients undergoing HD. A recent study reported severe critical Coronavirus disease 2019 (COVID‐19) in 41.9% of patients undergoing HD, compared to 15.8% in a control group without kidney disease.^1^

The most common and severe complication in patients with COVID‐19 is acute respiratory distress syndrome (ARDS), requiring admission to ICU for oxygen and ventilation therapies. Ninety‐four percent of patients with COVID‐19‐related ARDS need ICU stay for invasive mechanical ventilation (IMV),^2^ with possible long‐term endotracheal intubation and subsequent tracheotomy to facilitate ventilator weaning.^3^ Median ICU stay for patients with COVID‐19 varies widely between countries, ranging between 4 and 20 days.^4^ These ICU manoeuvres can lead to several complications as laryngotracheal granulomas, webs, stenosis, malacia and, less commonly, tracheal necrosis with tracheo‐oesophageal or tracheo‐arterial fistulae.^5^

We relate a fatal outcome of a post tracheotomy stenosis (PTS) as a complication of prolonged mechanical ventilation requiring tracheotomy, in a patient undergoing haemodialysis with COVID‐19‐related ARDS.

## CASE REPORT

A 44‐year‐old female patient was admitted to our ICU for a COVID‐19‐related ARDS and required IMV. The patient was known for a history of chronic HD for ESRD, obesity (BMI 35 Kg/m^2^), arterial hypertension, mechanical ventilation after cardiac arrest secondary to a severe cardiac arrhythmia and carried an automatic internal cardiac defibrillator.

After 17 days of intubation for IMV, prone ventilation during several days and systemic treatment with Dexamethasone, several attempts of weaning failed, hence percutaneous tracheotomy was performed by the Ear—Nose—Throat specialist (ENT) in the ICU. There was no evidence of airway disease when the tracheotomy was performed but the otolaryngologist noted an important bleeding when the trachea was opened suggesting important inflammation of the trachea.

The tracheotomy cannula was changed for the first time, 1 week later. This cannula was finally removed 8 days later, due to a satisfying improvement of the respiratory function. After a total stay of 32 days in the ICU, the patient was transferred to a COVID‐19 middle‐care unit for a week then to a rehabilitation centre, where she showed some improvement, but after 2 weeks she decided to return home and continue ambulatory HD.

During the following HD sessions, the patient complained of a persistent shortness of breathing and stridor, with an oxygen saturation varying from 95% to 98%. Laboratory findings were unremarkable. A chest CT scan was performed and showed no pulmonary embolism and no significant amelioration of the COVID‐19 lesions.

**FIGURE 1:**
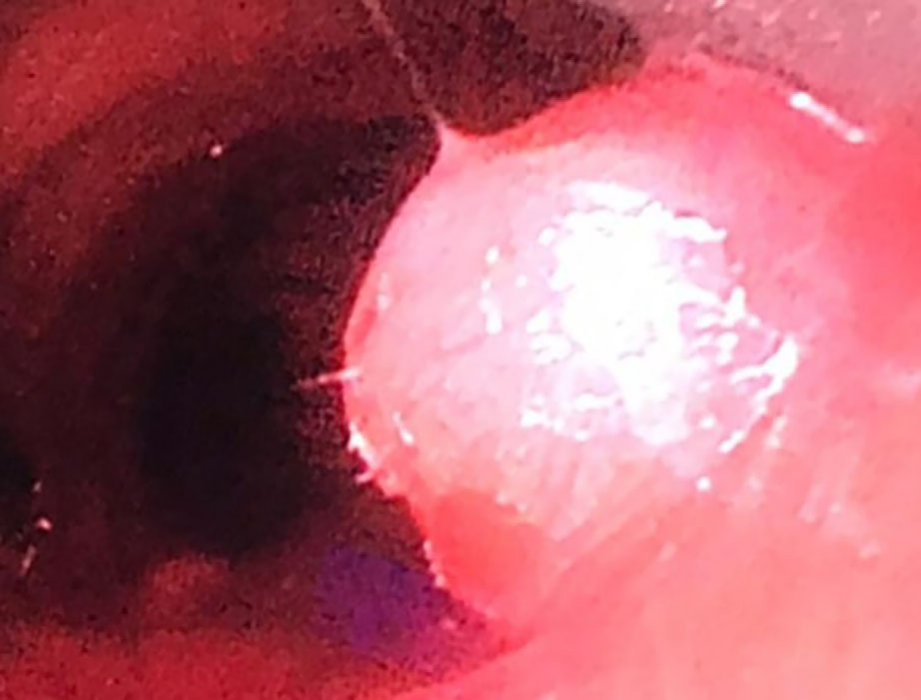
Fibro‐laryngoscopy showing one posterior subglottic granuloma.

Laryngoscopy was performed and showed a hard synechiae on the floor of the right nasal cavity, small lymphoid hyperplasia at the roof of the cavum and synechiae in the cavum was also observed. Oropharynx showed no particularities. The vocal cords were thickened, one small posterior subglottic granuloma was seen (Figure 1), subglottic area seemed slightly swollen and limitation of the vocal folds (VF) mobility in abduction as well as VF and subglottic edema was observed. At this point, the ENT specialist suggested close monitoring to detect signs of early tracheal stenosis. A bronchoscopy was then scheduled 2 weeks later.

Systemic Dexamethasone was administered to our patient first due to several weaning failures and then as prolonged administration because of persistent slight stridor. She also received inhalation aerosols namely Combivent® (ipratropium bromide and albuterol sulfate) and Pulmicort (Budesonide)® besides regular daily respiratory physiotherapy.

**FIGURE 2:**
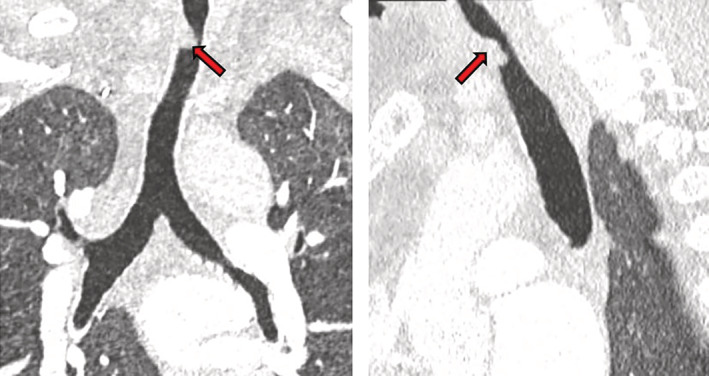
Axial and coronal CT scan views. Excess granulation tissue formation around the tracheal stoma site (arrow), responsible of a tracheal stenosis estimated to 40%.

**FIGURE 3:**
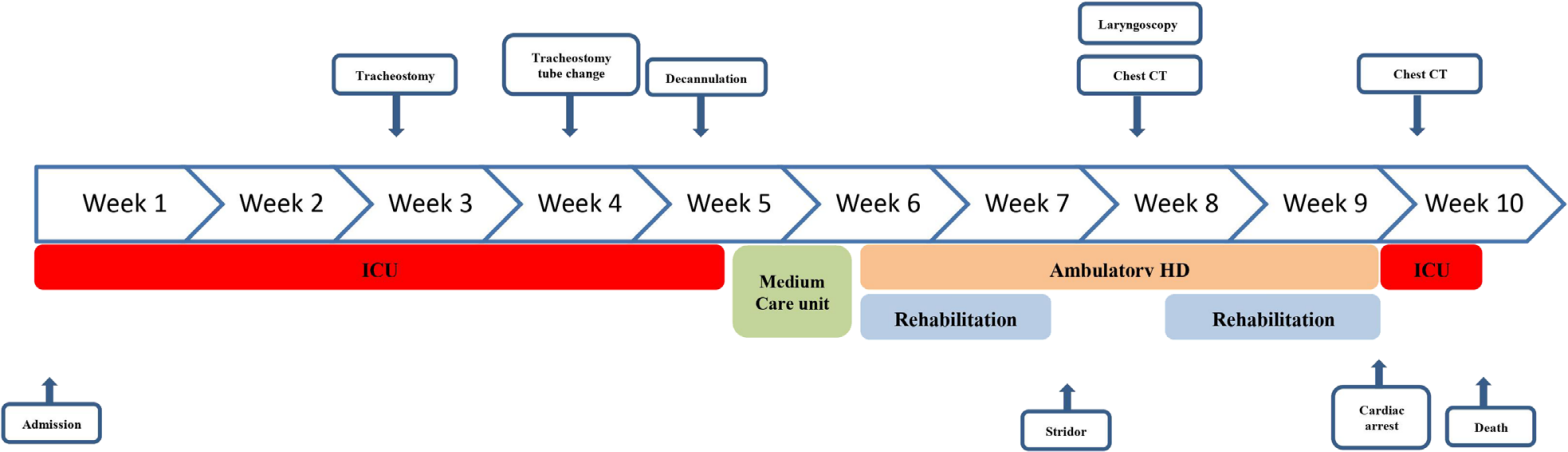
Time line of clinical course, management, and outcome.

Because of persistent functional problems such as fatigue, sleep dysfunction, chronic pain, shortness of breath and persistent stridor, the patient was re‐admitted to the rehabilitation centre, where she presented acute dyspnea, aggravation of stridor, severe hypoxemia and cardiac arrest. She was intubated and re‐admitted to the ICU. According to the resuscitation team, intubation was difficult. A new CT thoracic scan showed excess granulation tissue formation around the tracheal stoma site responsible for 40% tracheal stenosis (Figure 2). Without sedation, the patient remained comatose after 72 h and multiple prognostication tests done at this point, showed irreversible hypoxic‐ischemic brain injury. No postmortem examination was done due to refusal by the family and her community members. The clinical course and management of our patient is described in timeline figure (Figure 3).

## DISCUSSION

The most common cause of tracheal stenosis (TS) is iatrogenic, due to prolonged intubation or post tracheotomy or post tracheostomy.

The incidence of iatrogenic tracheal stenosis was much higher in the past. However, the use of endotracheal tubes with low‐pressure/high volume cuffs, did reduce the occurrence of this complication^6^ Nowadays, post‐intubation or post tracheotomy tracheal lesions are considered as an uncommon iatrogenic complication. Its incidence is extremely variable in the literature.^7^ It has been estimated to 4.9 cases per million of population per year, while a higher incidence has been reported in other studies, ranging from 1% to 21% of the intubated ICU patients.^8^

The incidence of TS seems to be much higher in patients with COVID‐19. The real incidence of TS in these patients could be much higher as a number of post COVID‐19 patients may not be referred for tracheal stenosis due to more debilitating COVID‐19‐related sequelae.^6^

TS of our patient was estimated to 40%, it was directly related to the tracheotomy as it was near the tracheotomy site. Performing a tracheotomy in a context of severe tracheal inflammation promotes tracheal stenosis. This tracheal inflammation is mainly related to the prolonged intubation and the delayed tracheotomy. Because of the high risk of accidental decannulation during prone position and the chance of cross‐infection of healthcare professionals, tracheotomy was postponed in patients with COVID‐19 needing IMV, until they no longer need to be ventilated in the prone position and had been determined to be cleared the virus. This practice is opposite to the usual non‐ pandemic standards, as tracheotomy performed after 7–14 days from endotracheal intubation significantly improved the chance of successful weaning and lowered the risk of complications and mortality when compared to long‐term maintenance of the orotracheal tube in place.^5^

Otherwise, multiple factors predisposing for post intubation or post tracheotomy laryngotracheal stenosis have been suggested by Al Turk et al. including: advanced age, female and oestrogen effect, smoking, obesity, and diabetes. Patients with peri‐tube infections, high tracheotomy site, traumatic intubation, history of previous intubation or previous tracheotomy, are also at high risk for TS.^9^

Our patient was at high risk for developing TS mainly because of prolonged intubation period and prolonged time before decannulation post tracheotomy. In addition, she had several other risk factors including sex, obesity, diabetes, previous intubation and important bleeding during tracheotomy. Her tracheotomy site was not high and she did not have peri‐tube infections that could explain her tracheal stenosis.

Severe inflammatory status associated with COVID‐19, could have also promoted the formation of granulation tissue and plugs. In addition, viral induced laryngeal and tracheal inflammation can be a distinct possibility for further upper airway damage.^10^, ^11^ Fiachini et al. compared patients under IMV for more than 14 days, in COVID‐19 infected and non‐COVID‐19 patient. The results showed increased incidence (47%) of tracheal lesions or tracheoesophageal fistulas in the COVID‐19 group compared to patients without COVID‐19, with only 2.2% incidence.^12^ One case of tracheal stenosis in a mild COVID‐19 patient, without a history of endotracheal intubation has been reported.^13^

In Figure 2, we show the granuloma which was seen in the fibro‐laryngoscopy.

This granuloma was situated in the posterior subglottic region. The formation of this granuloma in our patient could be multifactorial due to irritation and aspiration during the prolonged intubation period. Also, as we indicated before our patient had a percutaneous tracheotomy (PT) that was performed in the ICU and this is an important risk factor for granuloma formation. In fact, the pathogenesis of granulation formation is multifactorial.

TS following (PT) can also occur because of cartilage damage Using excessive force and large tubes and dilators during the PT procedure may also result in tracheal damage and stenosis.^14^ PT insertion was not reported to be difficult and hence we did not suspect cartilage damage as a cause of tracheal stenosis in our patient.

Poor wound healing in patients with a tracheostomy or tracheotomy can also lead to stenosis and be a challenge for the clinicians. Pulmonary secretions may accumulate around de stoma as well as excess of granulation tissue that may lead to wound infection and poor wound healing.^15^ Also, nutritional problems as seen often in ICU patients may impair wound healing.^16^ Our patient did not have a tracheal stoma but a tracheotomy that healed without any infectious complications.

ESRD or major CKD is also a risk factor for TS. The accumulation of uremic toxins in ESRD patients, leads to a chronic inflammatory state, which negatively impact mechanisms of wound healing secondary to slowed rates of neovascularization and cell proliferation, impaired keratinization, delayed granulation and large epithelial gaps. Patients with CKD suffer increased rates of wound disruption as compared to those patients with a normal glomerular filtration rate.^17^

Management of TS is of upmost importance for the good outcomes of the patients. Studies have shown that dexamethasone, a long‐acting and potent corticosteroid, is suitable for preventing post extubation airway edema. Administration of multiple prophylactic doses of dexamethasone significantly decreases the incidence of post extubation stridor in adult patients at high risk to develop airway obstruction.^18^

Our patient complained from shortness of breath and mild stridor, 2–3 weeks after ICU discharge and underwent a laryngoscopy, which showed mild lesions with preponderance in the subglottic area. Hence, she was re‐admitted to rehabilitation centre and a bronchoscopy was scheduled in 2 weeks delay. The clinical evolution of our patient is consistent with the description of similar TS reported cases.^5^ These reports showed that the time of onset of TS varied from 28 days to 6 months after extubation.^9^

The treatment of post tracheotomy tracheal stenosis has always been challenging due to late presentation or delayed diagnosis.^14^ Treatment modalities for TS need a multidisciplinary approach utilizing bronchoscopy, laser, airway stents and tracheal surgery. Nikolaos Zias et al. suggest that interventional bronchoscopy should be the first approach in the treatment sequence of patients with TS and that this may be the only required treatment in the majority of cases.^19^

Unfortunately, in our patient the scheduled bronchoscopy was too late, because the patient presented a rapid aggravation of her stridor and had a cardiac arrest. The 2 weeks delay for bronchoscopy is longer than usual, as the COVID‐19 pandemic has posed a real challenge for the follow‐up of outpatients, many consultations and elective surgeries have been postponed, especially for interventions concerning the airways, which represented a high risk situation for all health care workers (surgeons, anesthesiologists and operating room personnel).

The 2 weeks delay for bronchoscopy was also explained by the persistence of respiratory symptoms, considering the patient as possibly contagious for SARS‐COV‐2, as the symptoms she presented were attributed COVID‐sequelae. In a number of case reports, it was highlighted that common COVID‐19 respiratory symptoms, as cough and dyspnea after discharge from the hospital, were initially misdiagnosed and later attributed to post‐intubation tracheal stenosis.^6^

After patient's extubation or decannulation a close follow up by an experienced staff (otolaryngologist or other airway specialist) is necessary for every patient with a history of COVID‐19‐related ICU stay, in order to early diagnose post intubation complications such as TS. A worsening of breath on exertion or at rest after an initial improvement, associated to hoarseness, stridor, dry cough, or swallowing problems, must alert practitioners and raise suspicion of TS.

## CONCLUSION

TS is a well‐known complication of tracheotomy and prolonged intubation. It is not clear if TS occurs more frequently in COVID‐19 patients, but it seems that COVID‐19‐related ARDS patients are at high risk of developing TS due to their comorbidities and the need for a prolonged intubation. Thus, COVID‐19 epidemic may lead to a serious increase in TS incidence. All practitioners must be aware that COVID‐19 patients are at high risk for TS and a close follow up must be ensured by an experienced team in order to prevent, to diagnose early and improve its outcomes. Hence, we recommend physicians to be aware of TS, investigate and exclude post tracheotomy tracheal stenosis in COVID‐19 survivors who have been intubated in the past and who present with persistent shortness of breath or stridor.

## CONFLICT OF INTEREST STATEMENT

None declared.

## ETHICS STATEMENT

The authors declare that appropriate written informed consent was obtained for the publication of this manuscript and accompanying images.

## Data Availability

The data that support the findings of this study are available from the corresponding author upon reasonable request.
